# Gut yeast diversity of *Helicoverpa armigera* (Lepidoptera: Noctuidae) under different dietary conditions

**DOI:** 10.3389/fmicb.2024.1287083

**Published:** 2024-05-02

**Authors:** Man Yu, Yang Li, Jingyuan Ji, Yonghui Lei, Yanfei Sun

**Affiliations:** ^1^College of Life Sciences, Shihezi University, Shihezi, Xinjiang, China; ^2^College of Life Sciences and Food Engineering, Shaanxi Xueqian Normal University, Xi’an, Shaanxi, China; ^3^Department of Plant Protection, College of Agriculture, Shihezi University, Shihezi, Xinjiang, China

**Keywords:** *Helicoverpa armigera*, gut yeast microbiota, culture-independent yeast, host diet, high-throughput sequencing

## Abstract

Yeast is one of the important symbiotic flora in the insect gut. However, little is known about the gut yeast in *Helicoverpa armigera* (Lepidoptera: Noctuidae) under various dietary conditions. The composition and function of the intestinal yeast community also remain unclear. In this research, we explored the composition of yeast microorganisms in *H. armigera* larvae under different feeding environments, including apple, pear, tomato, artificial diet (laboratory feeding), *Urtica fissa*, *Helianthus annuus*, and *Zinnia elegans* (wild environment) using high-throughput sequencing. Results showed that a total of 43 yeast OTU readings were obtained, comprising 33 yeast genera and 42 yeast species. The yeast genera with a total content of more than 5% were *Hanseniaspora* (36.27%), *Moesziomyces* (21.47%), *Trichosporon* (16.20%), *Wickerhamomyces* (12.96%) and *Pichia* (6.38%). *Hanseniaspora* was predominant when fed indoors with fruits, whereas *Moesziomyces* was only detected in the wild group (*Urtica fissa*, *Helianthus annuus*, *Zinnia elegans*) and the artificial diet group. After transferring the larvae from artificial diet to apple, pear and tomato, the composition of intestinal yeast community changed, mainly reflected in the increased relative abundance of *Hanseniaspora* and the decreased abundance of *Trichosporon*. Simultaneously, the results of α diversity index indicated that the intestinal yeast microbial diversity of *H. armigera* fed on wild plants was higher than that of indoor artificial feeding. PCoA and PERMANOVA analysis concluded that there were significant differences in the gut yeast composition of *H. armigera* larvae on different diets. Our results confirmed that gut yeast communities of *H. armigera* can be influenced by host diets and may play an important role in host adaptation.

## Introduction

The intestines of all metazoan contain an abundant number of commensal microorganisms (Shin et al., 2011; Douglas, 2018). In the long-term co-evolution, insects provide a stable living environment and necessary nutrients for gut microorganisms, while gut microorganisms are also conducive to the survival of insects, significantly impacting nutritional metabolism (Gong et al., 2020), growth and development (Lauzon et al., 2000; Yang et al., 2020), reproductive behavior (Yong et al., 2017), immune disease resistance (Johnston and Rolff, 2015; Su et al., 2019), and other life processes of insects, forming a mutually beneficial symbiotic relationship (Dillon and Dillon, 2004; Shapira, 2016). Gut microorganisms have been widely demonstrated to degrade exogenous toxins (Abdelgaffar et al., 2019; Yang et al., 2020) and affect the transmission efficiency of vector insects (Fukatsu and Hosokawa, 2002).

However, bacteria are not the only inhabitants of the insect gut (Gurung et al., 2019). Mycobiota, especially yeasts, have been shown to be associated with many insects. Yeasts dominate the flora of some insects and establish a symbiotic relationship with the host insects (Malassigné et al., 2021). It has been found that yeast or yeast-like symbionts in insect tissue are involved in amino acid and fatty acid metabolic pathways, and yeast deficiency leads to the instability of host ecosystem regulation and incomplete metamorphosis (Carvalho et al., 2010; Horgan et al., 2021). Insects also lack the ability to synthesize sterols, multivitamins, and enzymes that degrade plant cell wall materials and toxic plant equivalents (Martin, 1979). This deficiency can be compensated for by intestinal symbiotic yeasts (Vega, 2005). For example, in the ground-dwelling beetle family *Silphidae*, whose species consume animal carcasses (Scott, 1998), the yeast *Yarrowia lipolytica* and a related species group capable of breaking down diverse carbon sources, including hydrocarbons and lipids (Miller and Alper, 2019), have been isolated (Kaltenpoth and Steiger, 2014). Vogel et al. also suggested that *Yarrowia* species help inhibit the growth of other microbes by producing antimicrobial substance (Vogel et al., 2017). Therefore, similar to bacteria (Yuan et al., 2021), gut yeast may be the linchpin of the survival mechanism of insects on hosts.

*Helicoverpa armigera* (Lepidoptera: Noctuidae) is a worldwide pest known to infest over 200 host plants, including cotton, tomato, pepper, potato, and various other crops and wild plants (Zheng et al., 2022). It occurs frequently in large crop-growing countries such as China, India, and Australia. Cotton bollworm larvae mainly eat the buds, flowers and fruits of host plants. After the flower is damaged, the plant cannot be pollinated normally, resulting in reduced production of cash crops such as tomatoes and cotton and greatly reduced the commodity fruit (Tsafack et al., 2013; Gu et al., 2022). Due of its wide distribution, omnivorousness, fast reproduction and strong adaptability (Fitt, 1989), *Helicoverpa armigera* has caused huge economic losses to global agriculture (Gujar et al., 2021). In 1992, the cumulative occurrence of cotton bollworm in various crops in China reached 21.92 million hectares, causing direct economic losses of 6 billion yuan. The widespread use of conventional insecticides could lead to many problems, for examples, resistance to conventional insecticides, environmental pollution, human health impacts, and injury to beneficial insects. In order to reduce the economic losses caused by cotton bollworm, countries worldwide began to promote the cultivation of genetically modified crops that express the insecticidal protein of *Bacillus thuringiensis* (Bt). By 2012, the global area of genetically modified crops had reached 191.7 million hectares (James, 2013). However, with the large-scale cultivation of genetically modified crops, the frequency of resistance in cotton bollworm has significantly increased worldwide (Shelton et al., 2002; Jin et al., 2015; Ahmad et al., 2019). Research indicates that the development of cotton bollworm resistance is largely attributed to internal microorganisms and indigenous gut bacteria increasing the tolerance of larvae to *B. thuringiensis* toxin (Takatsuka and Kunimi, 2000; Dubovskiy et al., 2016). In addition, the resistance of larvae to the entomopathogenic bacterium *B. thuringiensis* is affected by the maturity of the host plants. Martemyanov’s study showed that the negative effects of *B. thuringiensis* were eliminated when the larvae fed on mature leaves (Martemyanov et al., 2016). Gut microbial communities play a crucial role in the evolution of insect populations resistant to Bt toxins (Paramasiva et al., 2014). Therefore, gut microorganism is an important entry point to reveal the adaptation mechanism between different hosts of *H. armigera*.

The composition of the gut microbiota of insects can be affected by many factors, such as host species, genotype, diet, and the host living environment. Diets with different nutritional compositions can affect the longevity of insects (Edgar et al., 2011) and diet is one of the most prominent factors in the formation of intestinal microbial communities (Harris et al., 2019; Moran et al., 2019). But as far as we know, when *H. armigera* feeds on different fruits and flowers, there are no reports on the composition of gut fungal community, especially the community of gut yeast. To explore the impact of diets on the gut yeast communities of larvaes of *H. armigera*, we study the composition of intestinal yeasts of *H. armigera* fed on artificial feed, apple, tomato, pear (laboratory feeding), *Helianthus annuus*,*Urtica fissa* and *Zinnia elegans* (wild environment) by sequencing technology based on D1/D2 domains of the large subunit rRNA gene (26S rRNA). The results revealed the changes of intestinal yeast community in different hosts, and speculated the potential relationship between *H. armigera* and intestinal yeast community, which provided a new idea for the prevention and control of *H. armigera* in agriculture.

## Materials and methods

### Insect sample acquisition

Laboratory-reared cotton bollworms were kept in the artificial feed, at 26 ± 0.5°C, 70 ± 10% relative humidity (Rh), and 15H: 9H photoperiod (L: D) for more than 3 generations at the College of Agriculture, Shehezi University (Yuan et al., 2021). The composition of the artificial feed is shown in Table 1. The fourth-generation eggs were fed artificial feed to the first instar larvae stage, and then transferred to three different fruits (apple, pear, and tomato) to the third instar larvae stage. Cotton bollworms that continued to be fed artificial feed were used as a control group. See Supplementary material 1 for specific feeding processes. Three host fruits were collected from the Botanical Garden of Shihezi University in 2021. Wild third instar larvae were collected from a habitat in Shihezi City (N:44°15′23.14″ E:86°03′9.68″). The larval host plants are *Urtica fissa, Helianthus annuus,* and *Zinnia elegans,* respectively.”

**Table 1:** Experimental material information.

Samples	Source	Hosts	Note
UF	Wild environment	*Urtica fissa*	No
S	Wild environment	Sunflower (*Helianthus annuus*)	No
Z	Wild environment	*Zinnia elegans*	No
CK	Laboratory feeding	artificial feed	Cornmeal 220 g, Yeast powder 50 g, Glucose 35 g
P	Laboratory feeding	Pear	Korla Fragrant Pear
T	Laboratory feeding	Tomato	*Lycopersicon esculentum* var. *cerasiforme* A.Gray
A	Laboratory feeding	Apple	*Malus pumila Mill*

UF, S, Z, CK, A, T, and P represent the hosts of *H. armigera*, where A represents apple; P, pear; T, tomato; CK, artificial diet; UF, Urtica fissa; S, Sunflower; and Z, Zinnia.

### DNA extraction

Third instar larvae were first disinfected with 75% ethanol for 60 s, then with 0.5% sodium hypochlorite for 30 s, and finally washed with sterile water three times for 60 s (Wang et al., 2020). Guts were dissected with sterile clamp and washed again in sterile phosphate buffer saline (1 × PBS; pH:7.4). Thirty guts per treatment were collected into sterile centrifuge tubes (Anand et al., 2010), with three replicates per treatments. The FastDNA Spin Kit for Soil (MP Biomedicals, UAS) was used to extract the total DNA of samples.

### PCR amplification and high-throughput sequencing

Yeast 26S rRNA was amplified using a pair of specific primers: NL1F (forward primer) (5′-GCATATCAATAAGCGGA GGAAAAG-3′) and NL2R (reverse primer) (5′-CTTGTTCGCTATCGGTCTC-3′) (Keidser et al., 2012). The target DNA bands were amplified on a PCR thermocycler (ABI, California, CA, United States) with an initial denaturation at 98°C for 5 min, followed by 30 cycles of denaturation at 98°C for 30 s, annealing at 52°C for 30 s, and extension at 72°C for 45 s. Final extension was set at 72°C for 45 s. PCR products were analyzed using 1.8% agarose gel electrophoresis and finally purified using the AxyPrep DNA Gel Extraction Kit (Axygen Biosciences, USA). Quantification of the target fragment was performed using the QuantiFluor ™-ST (Promega, USA) (Zhang H. et al., 2018). The Illumina MiSeq PE300 platform (Illumina, USA) was used to perform paired end sequencing (2 × 300) of qualified libraries according to the instructions of Meiji Biomedical Company (Shanghai, China).

### Statistical and bioinformatics analysis

The software Trimmomatic (version 0.33, Golm, Germany) was used to filter the quality of the original sequence file, and then the software cutadapt (version 1.9.1, TU Dortmund, Germany) was used to identify and delete primer sequences with high quality readings without primer sequences (Bolger et al., 2014). Through FLASH (version 1.2.7, Baltimore, MD, USA) software, the high-quality readings of each sample are spliced by overlapping, and the resulting splicing sequences are clean reads (Magoč and Salzberg, 2011). We use UCHIME (version 4.2) to identify and delete chimera sequences and obtain the final valid data as a valid read. According to the sequence similarity, the effective Operational Taxonomic Units (OTUs) were clustered at 97% similarity level by software USEARCH (version 10.0) (Edgar et al., 2011). The classification of each D1 domain of the LSU (Ribosomal Large Subunit) rRNA sequence was analyzed by the Ribosomal Database Project (RDP) classifier algorithm (version 2.2). The confidence threshold used in the NCBI database (National Center for Biotechnology Information) was 0.7. Next, we plotted sparse curves to observe the community abundance and sequencing data of each sample (Li et al., 2018). Principal Coordinate Analysis (PCoA) was performed based on Bray Curtis at the OTU level, and the ‘vegan’ and ‘ape’ packages in R (version 4.0.2) were used to analyze the similarity or difference of sample community composition (Schloss et al., 2009). Difference tests between groups in PCoA were analyzed using Analysis of Similarities (ANOSIM) using the vegan package in R. Alpha diversity (CHAO1 estimator, ACE index, Shannon diversity index, and Simpson index) and yeast abundance were analyzed using SPSS 26.0 (IBM, NY, USA) (Zhu et al., 2021). All values are expressed as mean ± SE. When *p* < 0.05, the difference is considered significant, and when *p* < 0.01, the difference is considered extremely significant.

## Results

### Analysis of 26S rRNA high-throughput sequencing

**Figure 1:**
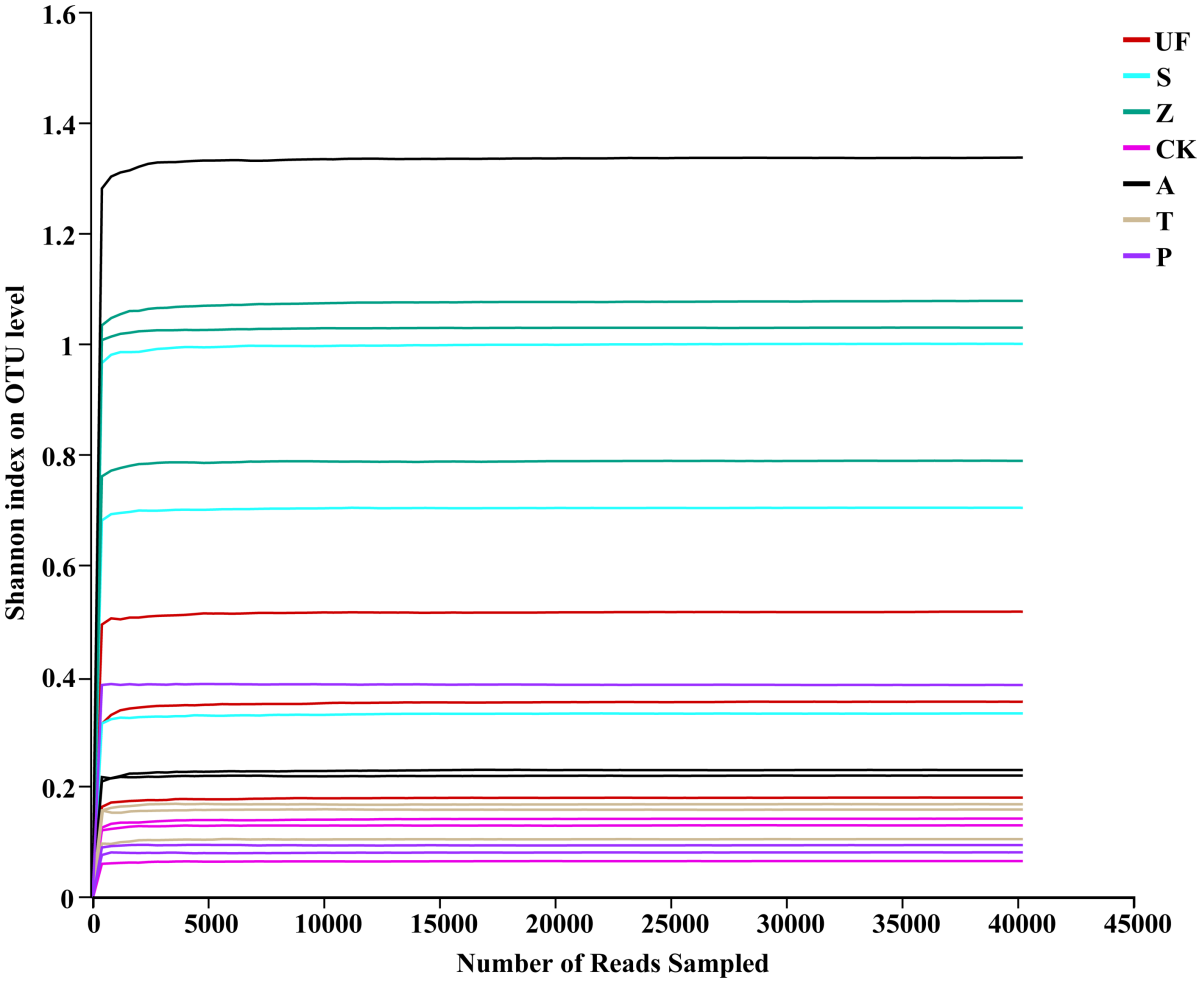
Rarefaction curves of insect gut from different samples. Rarefaction curves of OTUs were clustered for a dissimilarity threshold of 3%. Each sample had three replicates (Replicates are not specifically shown in the legend, but have been involved in the analysis). Samples abbreviations are as in Table 1.

Illumina HiSeq technology was used to sequence 21 samples from 7 treatments. Following splicing and filtering, a total of 1,296,261 clean tags were generated, with an average of approximately 62,727 clean labels per sample. Through cluster analysis based on 97% sequence similarity, we identified 224 OTUs, including 22 phyla, 47 classes, 92 orders, 130 families, 164 genera, and 191 species. Specifically focusing on yeast, we identified 40,703 total reads and 43 OTUs, spanning 2 phyla, 9 classes, 12 orders, 20 families, 33 genera, and 42 species. The sample Shannon index dilution curve (Figure 1) shows that the sequencing depth is saturated, suggesting that the sequencing amount is sufficient and increasing the sample size will not produce more OTUs.

### Comparison of intestinal gut yeast groups of *Helicoverpa armigera* under different hosts

The α diversity index (Table 2) showed that there were differences in the community structure of intestinal yeasts in *H. armigera* fed on different diets. The abundance of yeasts in the intestinal tract of *H. armigera* fed on wild plants (UF, S, Z) was notably higher than that of the laboratory artificial fruit feeding group (A, T, P). Particularly, the abundance of yeasts in the intestinal tract of wild *H. armigera* fed on *Zinnia elegans* (Z) was significantly higher to the laboratory artificial feeding group (CK, A, T, P), with a Chao 1 index of 17.17 ± 4.37. The richness of UF, S, and Z groups was similar, with no significant difference between CK, A, T, and P groups. According to the difference analysis between groups, the Shannon index of the S group was the highest, indicating significantly higher yeast diversity compared to the A and P groups. However, the diversity of the gut yeast population did not change significantly when the cotton bollworm was transferred from artificial feed to apple, tomato, and pear.

**Table 2:** Alpha diversity indices of yeast in insect gut from different samples.

Sample ID	Chao1	ACE	Shannon	Simpson
UF	12.37 ± 3.90ab	15.46 ± 4.22ab	1.27 ± 1.10ab	0.40 ± 0.51ab
S	12.33 ± 2.89ab	12.83 ± 1.63abc	1.95 ± 0.10a	0.15 ± 0.06b
Z	17.17 ± 4.37a	18.62 ± 3.67a	0.66 ± 0.33b	0.72 ± 0.13a
CK	7.83 ± 3.33b	8.45 ± 3.26bc	1.21 ± 0.22ab	0.40 ± 0.11ab
A	10.42 ± 1.51b	6.69 ± 5.89c	0.69 ± 0.27b	0.65 ± 0.16**a**
T	9.00 ± 1.73b	16.40 ± 7.82ab	1.13 ± 0.14ab	0.40 ± 0.03ab
P	8.33 ± 0.29b	14.23 ± 5.52abc	0.41 ± 0.16b	0.80 ± 0.13a

Each sample had three replicates. Sobs index was the observed species richness, Chao1 and ACE indices were used to evaluate species richness, Shannon and Simpson indices were used to evaluate species diversity. Larger Simpson index values indicate lower species diversity. The values of mean ± SE (standard error) of three samples are shown in the table. The different lowercase letters show the significant difference of the same index between different treatment samples (Kruskal-Wallis test, *p* < 0.05). Samples abbreviations are as in Table 1.

**Figure 2:**
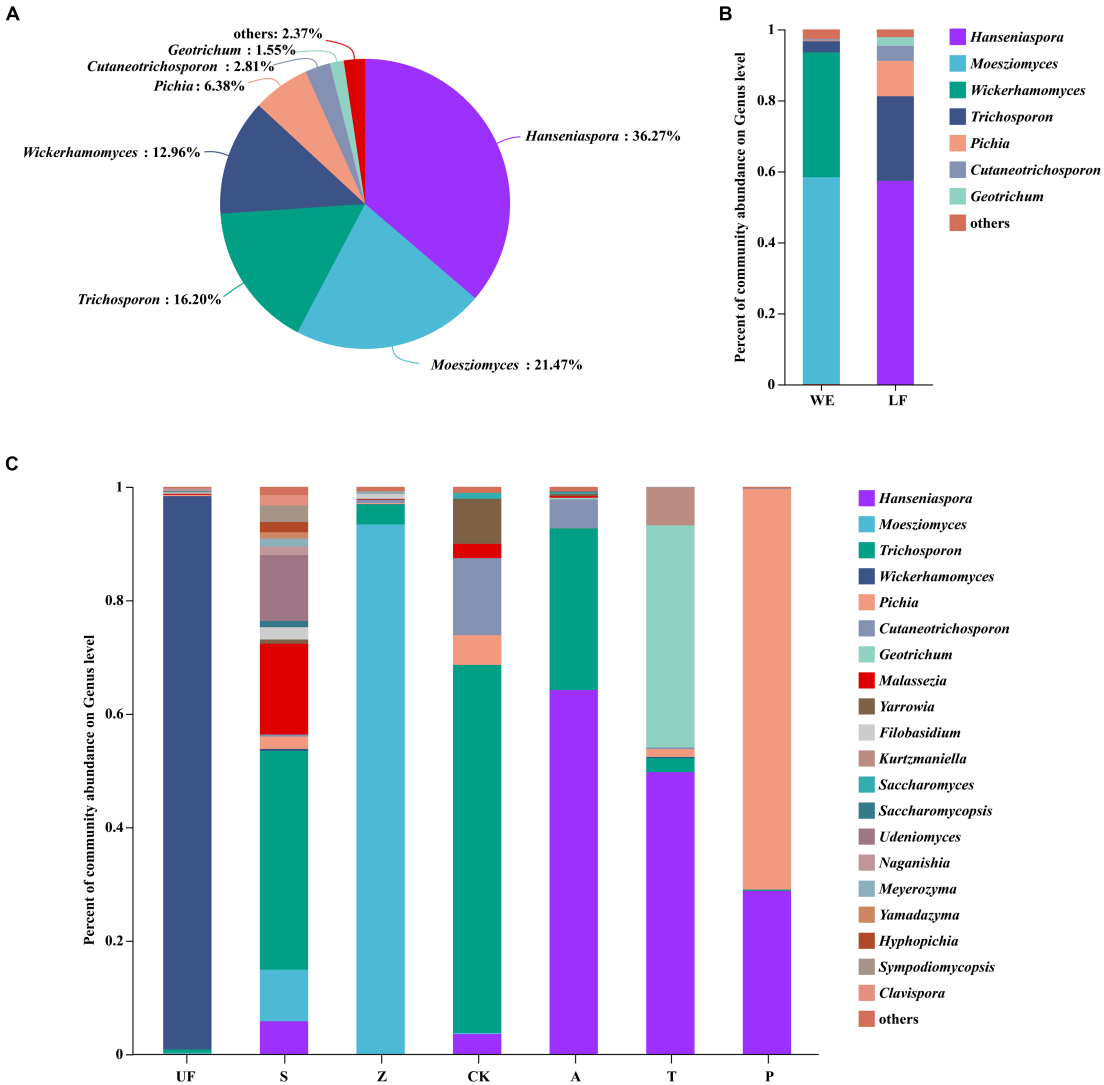
Each sample had three replicates. **(A)** Yeast community analysis diagram of all samples at the genus level. **(B)** Relative abundance of yeast in laboratory feeding samples (LF includes A, T, and P samples) and wild environmental samples (WE includes UF, S, Z samples) at the genus level. **(C)** Relative abundance of yeast at the genus level for each sample.

After quality filtering, the representative sequences of OTUs were compared with the NCBI reference database to obtain species classification information corresponding to each OTU. Subsequently, the composition of each sample was quantified at both the genus and species levels. At the genus level (Figure 2A), *Hanseniaspora* (36.27%), *Moesziomyces* (21.47%), *Trichosporon* (16.20%), *Wickerhamomyces* (12.96%) and *Pichia* (6.38%) exhibited total relative abundance greater than 5%.

*Hanseniaspora* is an absolutely dominant genus, and its relative abundance in artificial feeding samples is generally higher than that in wild sampling groups. The highest relative abundance was observed in the apple feeding group (A) with a value of 77.05 ± 14.18%. The other three groups are: artificial feed feeding group (CK), with a relative abundance of 3.97 ± 6.34%; tomato feeding group (T) at 44.54 ± 11.64%; and pear feeding group (P) at 68.78 ± 41.66%. However, this genus was not detected in the UF group. *Moesziomyces*, on the other hand, shows lower relative abundance in the artificial feeding samples compared to wild sampling groups. Its highest relative abundance in the Z groups was 90.45 ± 10.94%, whereas it was not detected in the artificial feeding groups (A, T, P). The relative abundance of *Trichosporon* was higher in the apple feeding group (A) and the feed feeding group (CK). *Wickerhamomyces* exhibits a relative abundance of more than 5% in the UF group, at 40.04 ± 51.68%, and the relative abundance of *Pichia* does not show significant differences among the seven groups. Notably, the relative abundance of *Hanseniaspora* significantly increases in other groups, while the relative abundance of *Trichosporon* decreases after feeding *H. armigera* from feed to different fruits (Figures 2B,C; Table 3).

**Table 3:** The relative abundance of the top five genus.

Sample ID	*Hanseniaspora*	*Moesziomyces*	*Trichosporon*	*Wickerhamomyces*	*Pichia*
UF	0.00 ± 0.00c	13.56 ± 17.50b	10.11 ± 17.41c	40.04 ± 51.68a	3.48 ± 6.01c
S	2.34 ± 4.05c	14.64 ± 10.94b	34.28 ± 6.30b	3.03 ± 5.25b	7.66 ± 7.0b
Z	0.04 ± 0.03c	90.45 ± 10.94a	1.51 ± 1.15c	0.00 ± 0.00b	0.01 ± 0.01c
CK	3.97 ± 6.34c	1.57 ± 2.75b	56.93 ± 12.62a	0.00 ± 0.00b	6.27 ± 10.85b
A	77.05 ± 14.18a	0.00 ± 0.00b	18.32 ± 11.24bc	0.00 ± 0.00b	0.27 ± 0.25c
T	44.54 ± 11.64b	0.00 ± 0.00b	3.37 ± 1.67c	0.57 ± 0.76b	2.35 ± 2.35c
P	68.78 ± 41.66ab	0.00 ± 0.00b	0.42 ± 0.23c	0.00 ± 0.00b	28.92 ± 43.32a

The values of mean ± SE (standard error) of three samples are shown in the table. The different lowercase letters show the significant difference of the same index between different treatment samples (Kruskal-Wallis test, *p* < 0.05). Samples abbreviations are as in Table 1.

**Figure 3:**
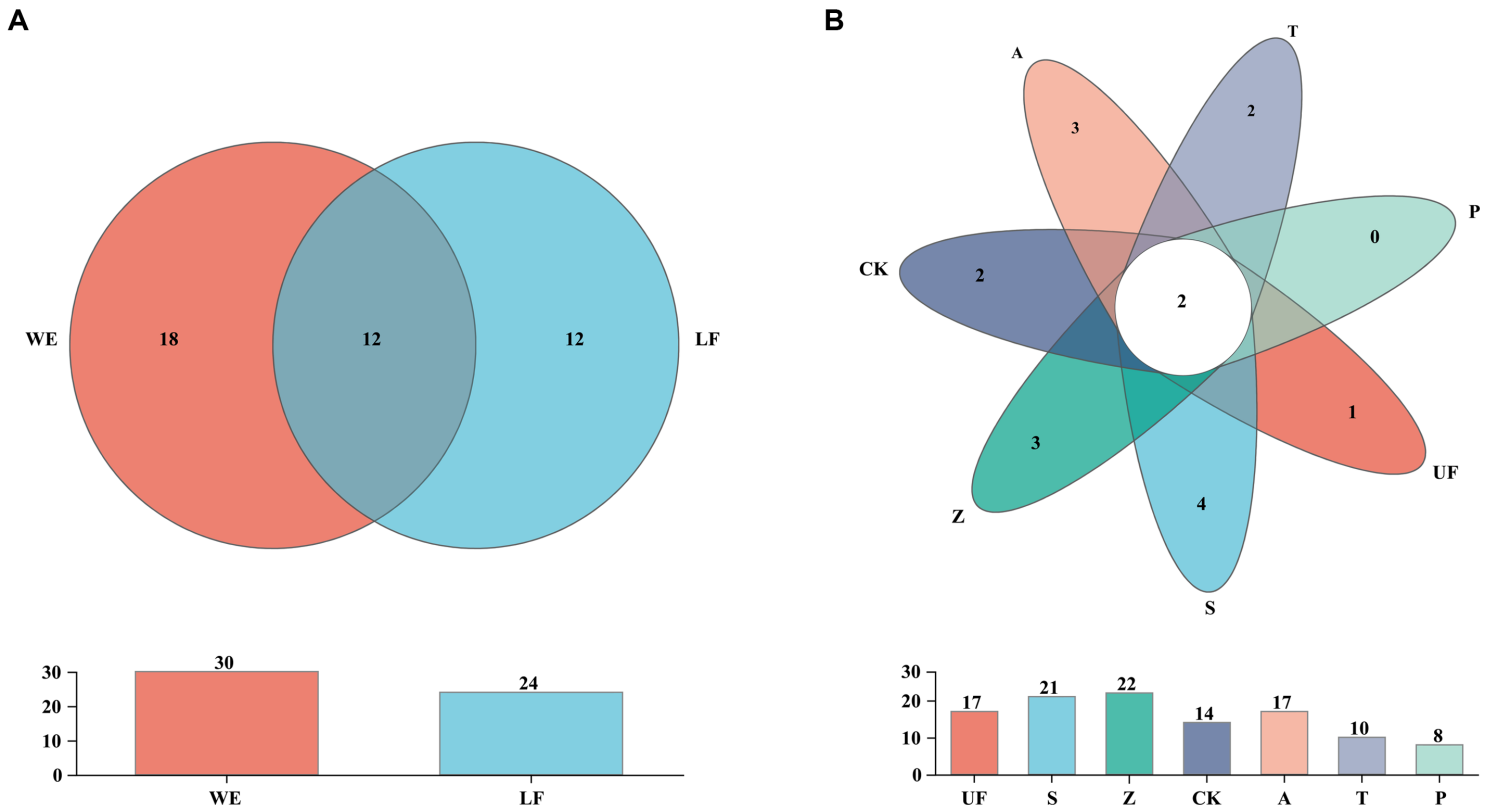
Venn diagram at the species level of samples. **(A)** Represents laboratory feeding sample (LF includes CK, A, T, and P samples) and wild environmental samples (WE includes UF, S, Z samples). **(B)** Represents each sample. Each circle with different colors in the diagram represents a group; middle core numbers represent the number of species common to all groups.

In order to count the number of species in multiple samples and the information of common and unique species, we drew a Venn diagram at the species level (Figure 3). A total of 52 species were detected in all intestinal samples. Further analysis revealed that there were 12 yeast species in wild and laboratory artificial samples (Figure 3A). We also found that each sample had its own unique yeast species. At the species level, group Z had the highest number of detected species, with 22 species, while group T had the lowest, with 10 species (Figure 3B). In all samples, *Trichosporon* sp. and *Cutaneotrichosporon curvatum* were common species, and three unique species-*Sympodiomycopsis* sp., *Pichia occidentalis*, *Clavispora lusitaniae,* and *Pichia terricola* appeared in sample S (Table 4).

**Table 4:** Exclusive and shared species in all samples.

Sample	Only species	All shared species
UF	*Metschnikowia pulcherrima*	*Trichosporon* sp. *Cutaneotrichosporon curvatum*
S	*Sympodiomycopsis* sp.	
	*Pichia occidentalis*	
	*Clavispora lusitaniae*	
	*Pichia terricola*	
Z	*Papiliotrema laurentii*	
	*Sporobolomyces roseus*	
	*Papiliotrema* sp.	
CK	*Malassezia* sp.	
	*Exophiala lecanii-corni*	
A	*Kondoa gutianensis*	
	*Candida saitoana*	
	*Kazachstania slooffiae*	
T	*Wickerhamomyces pijperi*	
	*Kurtzmaniella natalensis*	
P	No	

Samples abbreviations are as in Table 1.

**Figure 4:**
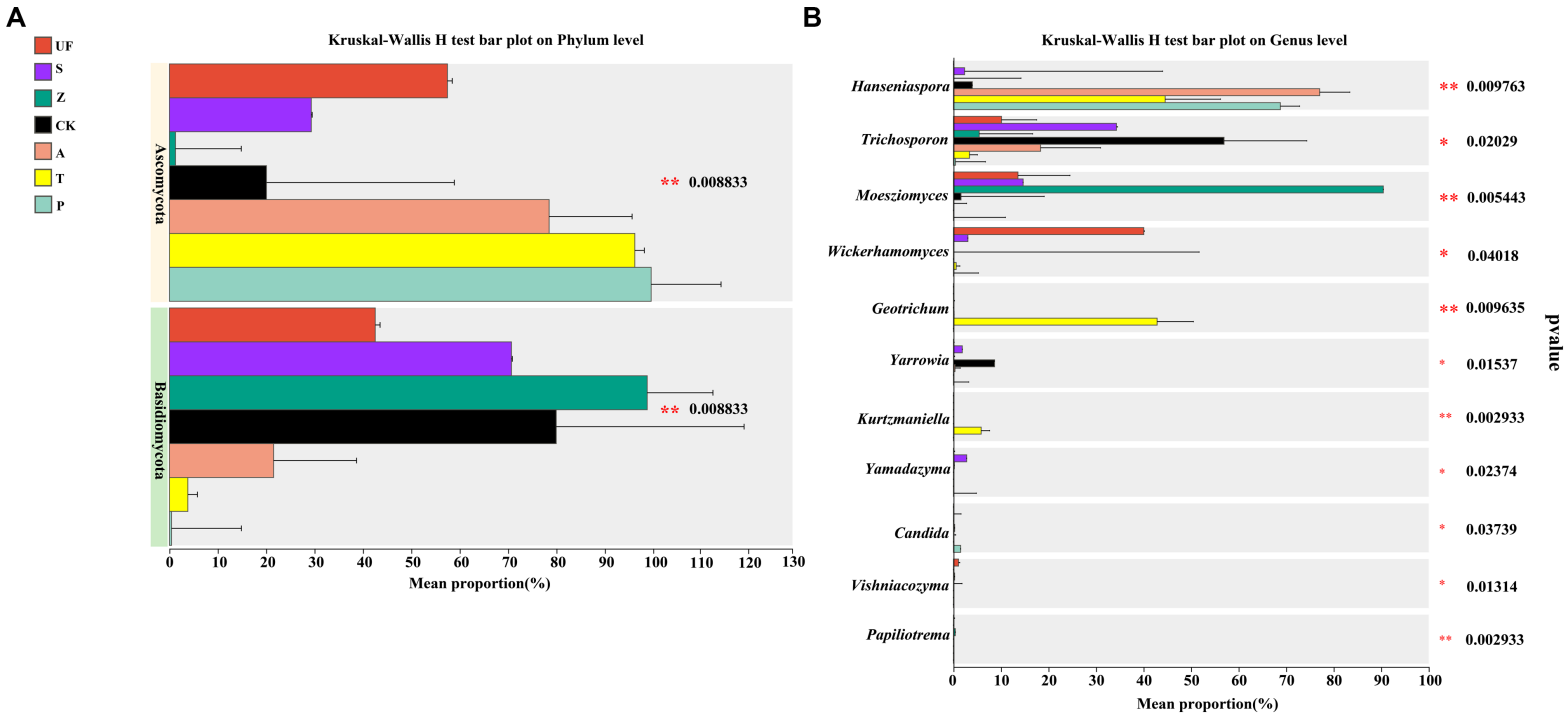
Species difference analysis of seven samples: **(A)** Phylum level; **(B)** Genus level. The y-axis represents the classification levels of species, and the x-axis represents the percentage of species average relative abundance in each sample group. Different colors represent different samples. The Kruskal-Wallis rank-sum test was used to show significant differences (*0.01 < *p* < = 0.05, **0.001 < *p* < = 0.01, ***p < = 0.001).

The results of species difference analysis, based on the phylum level, showed that the yeast species in the intestinal samples of wild *H. armigera* were mainly concentrated in *Basidiomycetes*. In contrast, the laboratory feeding samples were predominantly composed of *Ascomycota* (Figure 4A). At the genus level (Figure 4B), there were significant differences in 6 genera (*p* < 0.05) and extremely significant differences in 5 genera (*p* < 0.01) among the 7 groups. *Moesziomyces* and *Trichosporon* mainly found in UF, S, and Z groups, while *Hanseniaspora* and *Trichosporon* were primarily detected in CK, A, T, and P.

**Figure 5:**
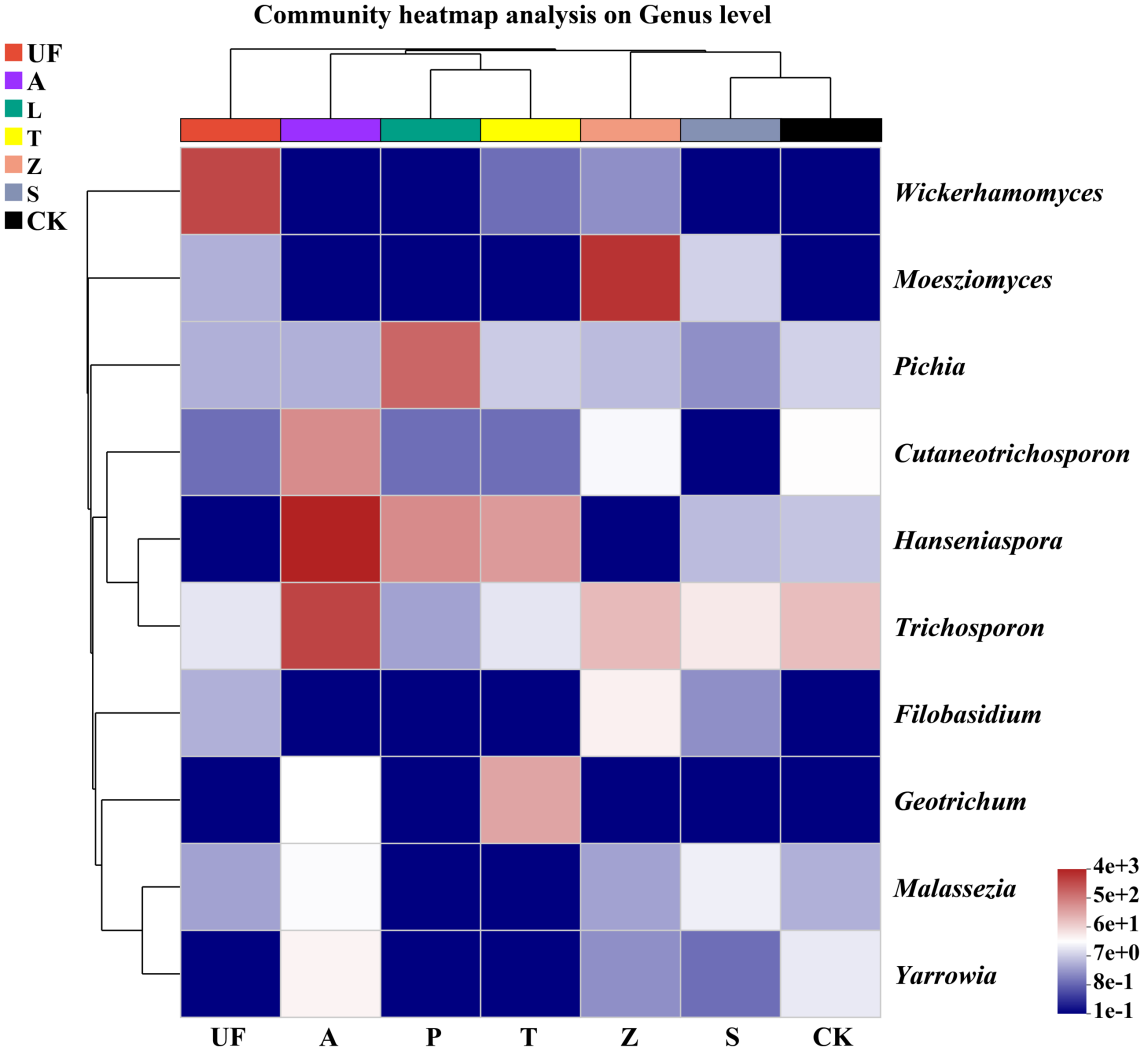
Cluster heat map of the 10 most abundant genera in the yeasts community. The columns represent the samples and the rows represent the yeasts OTUs assigned to the genus level. Dendrograms of hierarchical cluster analysis grouping genera and samples are shown on the left and at the top, respectively. Samples abbreviations are as in Table 1.

To elucidate the dynamic shifts in the intestinal microflora of *H. armigera* across various hosts, we selected 10 genera with the highest relative abundance for constructing a relative abundance cluster heat map. The clustering is based on the similarity in species abundance, with horizontal clustering representing sample information and vertical clustering indicating species information. Wild plants, including Urtica fissa (UF), Zinnia (Z), and sunflower (S), exhibit distinct clustering on separate branches. Notably, the gut yeast community compositions of *H. armigera* feeding on pear (P) and tomato (T) are highly similar. *Moesziomyces* emerges as a major component in the gut microbiome of *H. armigera* feeding on *Zinnia elegans*, while the relative abundance of *Wickerhamomyces* is notably higher in the gut of *H. armigera* feeding on Urtica than in other groups. Similarly, *Hanseniaspora* and *Trichosporon* exhibit elevated relative abundances in the A group (Figure 5).

**Figure 6:**
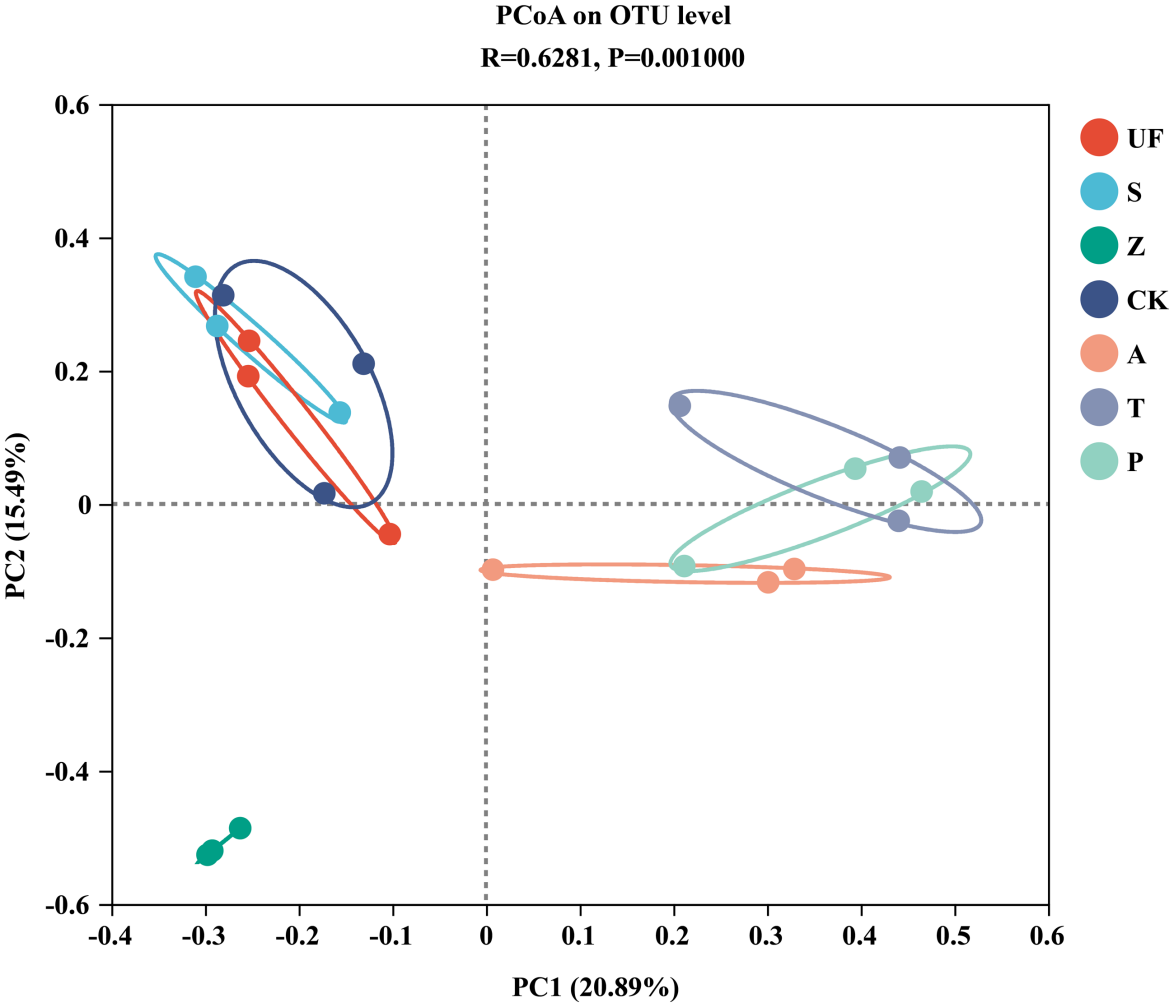
Principal Coordinates analysis (PCoA) based on Bray-Curtis distance method at the OTU level. Samples abbreviations are as in Table 1.

**Figure 7:**
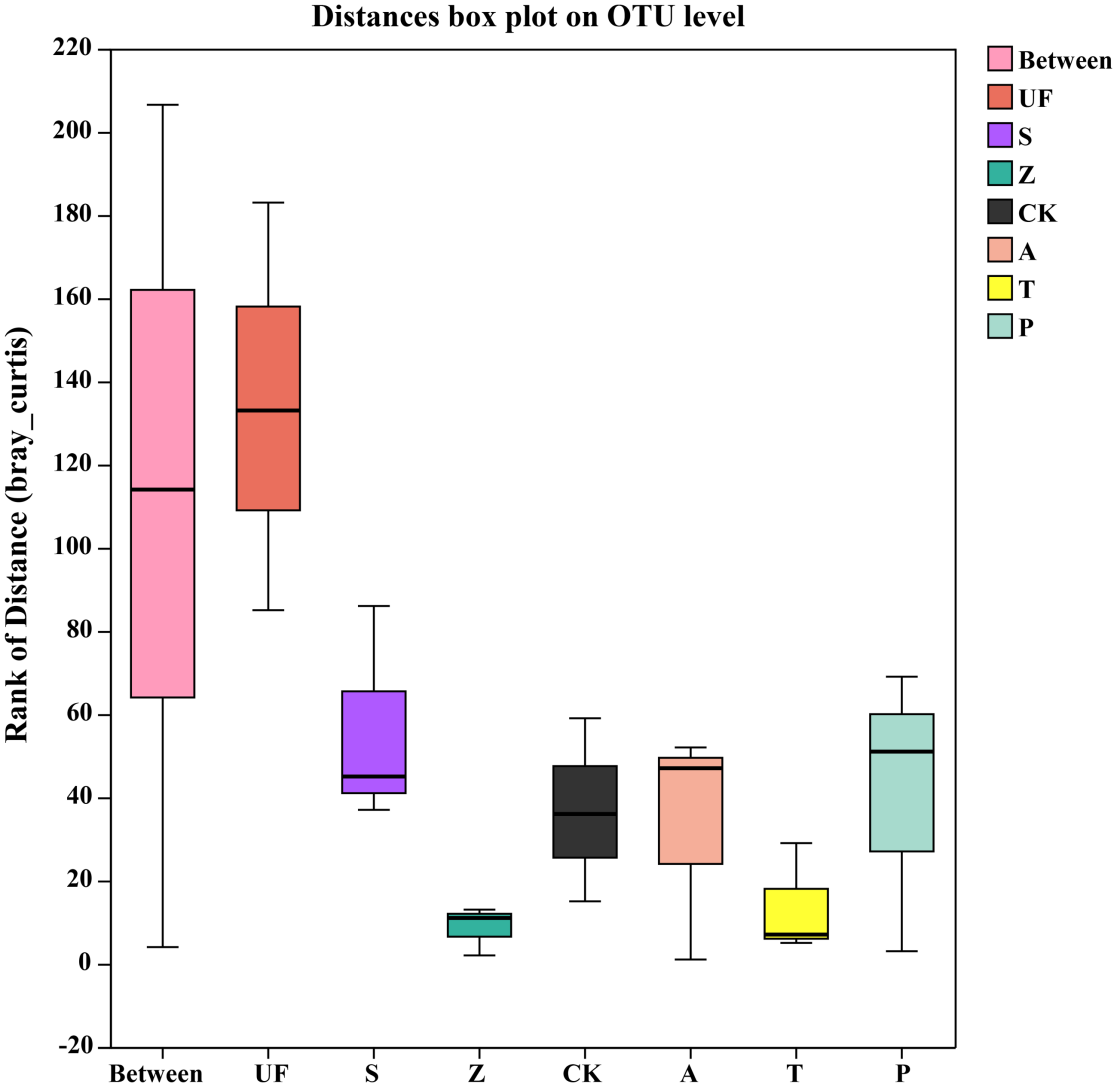
Adonis analysis account for the sample differences by different grouping factors. The ‘Between’ boxes refer to differences between groups, while the others represent differences within their respective groups.

Sorted at the OTU level by PCoA to compare community composition similarities between samples. The abscissa and ordinate, respectively, explain the two eigenvalues that contribute the most to the difference between the samples, and the influence degree is 20.89 and 15.49%, respectively, (Figure 6). PCoA1 has relatively small eigenvalues and captures less than 50% of the changes in the input data, so it is not considered to be a very successful PCoA. However, the results of Adonis analysis (Figure 7) showed that the R2 value (R2 = 0.6281, *p* = 0.001) was greater than 0 and tended to 1, indicating that the difference between the sample groups was greater than that within the sample group. Among the seven insect gut samples, most of the samples in each group clustered together, the three replicates of each sample had good reproducibility, and the degree of separation between each sample group was better, with an AP-value <0.05 (p = 0.001), indicating that there were significant differences in community composition between groups.

## Discussion

*Helicoverpa armigera* (Lepidoptera: Noctuidae) is an omnivorous and highly migratory agricultural pest. The hatched larvae feed on the tender leaves, flowers, or fruits of plants (Luo et al., 1999). The growth, development, and survival rate of the cotton bollworm are influenced by different host plants (Kim and Lee, 2002). In this research, we have studied the gut yeast diversity and community composition of *H. armigera* larvae feeding on different wild plants and artificial laboratory fruits by high-throughput sequencing. The experimental results provided a comprehensive understanding of the relationship between *H. armigera* and its symbiotic yeast. According to our experimental data, we can preliminarily conclude that the diversity and relative abundance of the gut yeast community of *H. armigera* are affected by the host diet. There is a complex yeast symbiotic community in the gut of *H. armigera* larvae, which provides a theoretical basis for comprehending the adaptive mechanism of *H. armigera* and its host plants.

Given the potentially crucial roles of microbial communities in insect physiology and development, extensive research has unveiled the diversity of microbial communities in insects (Douglas, 2007). Some studies indicates that the diversity of fungal communities in the insect gut is influenced by a range of complex factors such as the insect’s habitat environment, diet, developmental stage, and phylogeny. Unlike bacteria, fungi are more strongly influenced by environmental factors, such as different diets and geographical locations, than physiological constraints (Zhang Z. et al., 2018; Park et al., 2019; Ludvigsen et al., 2020). A survey of the gut fungal community of *Bactrocera tryoni* (Froggatt), Queensland fruit fly larvae feeding on different host fruits revealed that the yeast *Pichia* had a relative abundance of 90% in the larval gut when feeding on loquats and less than 1% relative abundance in the gut when feeding on pomegranates (Majumder et al., 2020). Insects from different geographical populations typically have distinct gut microbial communities. The gut fungal community of beetles (*Coleoptera*) is also influenced by their habitat. There are significant differences in the yeast community structure in the guts of beetles between forests and grasslands (Kudo et al., 2019). Reports on the gut microbiota of *H. armigera* have primarily focused on bacteria. The dominant bacterial phylum in the gut of *H. armigera* is *Actinobacteria* (Ranjith et al., 2016). The diversity of symbiotic bacteria in the gut of cotton bollworm larvae feeding on cotton is greater compared to those feeding on artificial feed (Zhao, 2021). In our study, variations in the yeast population composition were observed when cotton bollworm larvae were transitioned from artificial feed to pear, apple, and tomato diets. The yeast diversity in the gut of cotton bollworm larvae feeding on wild plants (UF, S, Z) surpassed that of the laboratory artificial fruit feeding group (CK, A, T, P). Particularly, the yeast diversity in the gut of cotton bollworm larvae feeding on *Zinnia elegans* was notably higher than that of the laboratory feeding group.

At the phylum level, the gut yeast population of cotton bollworm larvae consuming plants was predominantly composed of *Basidiomycetes*. In contrast, the gut yeast population of laboratory-fed cotton bollworm larvae consuming fruits was dominated by *Ascomycota*. At the genus level, *Hanseniaspora* was the predominant genus detected in *H. armigera* fed on fruits. This observation may be attributed to the fact that *Hanseniaspora* is the prevalent yeast genus in the early stages of natural fermentation, commonly found on the epidermis of mature fruits, and capable of secreting various hydrolases. Certain strains of this genus exhibit low ethanol production, facilitating insect digestion of the pulp without causing harm to the host insects (Capece et al., 2005; Viana et al., 2009; Hong and Park, 2013). *Moesziomyces,* belongs to the phylum *Basidiomycota,* is primarily distributed in wild larval samples (UF, Z, S), and is rarely detected in laboratory samples. This yeast genus was initially isolated from sediment in Lake Vanda, Antarctica (Li et al., 2019) and later frequently isolated from the surface of plant leaves, serving as a natural source for plant resistance against powdery mildew (Liu et al., 2019). *Trichosporon* exhibit relatively high abundance in artificial feed (CK), with a relative abundance similarly to *Wickerhamomyces* in the wild *Urticafissa* (UF) group. These results strongly suggest a certain correlation between the yeast populations in the cotton bollworm gut and those on the host, indicating potential similarities. The microbial community in the insect gut is influenced by the host’s feeding habits, leading to different dominant microbial lineages (Ayayee et al., 2016; Vilanova et al., 2016; Bing et al., 2018; Liu et al., 2020).

*Trichosporon* spp. and *Cutaneotrichosporon curvatum* were found in all seven treatment samples. *Trichosporon* spp. are versatile organisms that can thrive in diverse environments, including soil, wastewater, sediment, wood pulp, and sludge (Mehta et al., 2021). They have the capability to utilize their cells or enzyme systems for bioremediation, breaking down pollutants and external substances. This occurs particularly when using uric acid, ethylamine, aniline, and aromatic compounds as the exclusive energy source (Godjevargova and Gabrovska, 2003; Bergauer et al., 2005). Another species, *Cutaneotrichosporon curvatum,* exhibits the capacity to utilize unconventional carbon sources for oil production and is proficient in lipid accumulation (Péter et al., 2019). Several yeast species found in the wild samples display special function. *Metschnikowia pulcherrima* demonstrates the ability to produce various acidic proteases and microbial lipids (Li et al., 2021). Additionally, *Papiliotrema laurentii* in group Z stands out as an oleaginous yeast with a diverse range of metabolic carbon sources, enabling the production of enzymes and high lipid concentrations (de Almeida et al., 2022). Similarly, *Sporobolomyces roseus* can produce zeatin (Streletskii et al., 2019) and degrade amino acids (Moore et al., 1968). *Papiliotrema* has been reported to be resistant to heavy metals (Nguyen et al., 2020). These biological functions possibly supply essential nutrients for the growth of cotton boll larvae or aid the host in detoxification processes. Different yeast species appeared in the gut of cotton boll larvae under different feeding conditions. This difference could be a result of wild larvae adapting to the constantly changing natural environment, including factors such as sunlight, temperature, moisture, and food availability. The larvae may require a greater presence of symbiotic yeasts with specific functions to help them withstand external adversities.

PCoA analysis revealed variations in the composition of gut yeast populations. Interestingly, we observed no significant difference in the structure of intestinal yeast microflora between the groups fed tomatoes (T) and pears (P) (Permonova: R^2^ = 0.275, *p* = 0.3). A daring hypothesis to elucidate this observation is that tomatoes and pears share certain similar nutrients, potentially amino acids or acids, fulfilling the growth requirements of cotton bollworm larvae (Shao et al., 2011). It is well-established that the composition of gut microbiota is influenced by both plant secondary metabolites and the nutritional needs of the host (Myers et al., 2006; Blanco et al., 2007; Muhammad et al., 2017). Insects’ host are influenced by many factors, including environmental conditions, dietary maturity, host volatiles, and so on, all of which indirectly affect the composition of the insect gut symbiotic microbial community. There is a close relationship between the three (insect, insects host and the insect gut symbiotic microbial community).

Controlling *H. armigera* in China poses significant challenges, with current methods relying on chemical pesticides and the cultivation of Genetically Modified (GM) crops. However, people are gradually realizing that these methods confer stronger resistance to insects. Transgenic pest control strategies involving the modification of microorganisms (Paratransgenesis) have been proposed. In this strategy, genetically engineered microorganisms colonize the insect gut and produce effector molecules that target and kill pests. For instance, research has integrated genes expressing lytic peptides into yeast, creating an eco-friendly bait system targeting termites. The engineered yeast spreads widely in the social insect population, and the expressed lytic peptides kill protozoa in the gut within 4 weeks, followed by termite mortality within 6 weeks (Sethi et al., 2014).

Another innovative strategy is RNA interference (RNAi), a reverse genetic approach with potential applications in pest control. Yeast, commonly found on the surface of many fruits and constituting a major part of the microbial communities in various insect species, makes it highly attractive to insects. Murphy et al. (2016) utilized genetically modified baker’s yeast to feed *Drosophila suzukii*, delivering species-specific dsRNA. After feeding on this “yeast biopesticide,” the survival rate of *Drosophila* larvae, as well as the mobility and reproductive capacity of the adults, was reduced (Murphy et al., 2016). There are also relevant experiments using fungi as feeding stimulants to increase insecticide ingestion. For example, the combination of sucrose with *Saccharomyces cerevisiae* or *Aureobasidium pullulans* can serve as ingestion stimulants, enhancing the efficacy of Cyantraniliprole and Spinosad (Knight et al., 2015). Yeast can also serve as an adjuvant for biopesticides. Yeast isolated from the gut of *Cydia pomonella* larvae can enhance the activity of *Cydia pomonella* granulovirus (CpGV), significantly increasing the mortality rate of newly hatched larvae (Knight and Witzgall, 2013). Fungi that are attractive to insects can be directly used for pest monitoring, forecasting, and lure-and-kill strategies. For instance, the yeast *Candida utilis* has been used as bait to trap adult vinegar flies (Daane and Johnson, 2010).

In conclusion, our research findings confirm that the gut yeast community of cotton bollworm larvae is influenced by the host’s diet. Variations in dominant yeast species occur with different host diets, and transitioning cotton bollworm larvae from artificial feed to apple, pear, and tomato diets induces changes in yeast population abundance. These alterations likely contribute to mutual adaptation with the host. Understanding the composition of the gut yeast population in cotton bollworms is crucial due to the physiological and ecological functions of symbiotic yeast. This knowledge lays the foundation for developing targeted pest control technologies based on symbiotic yeast (Berasategui et al., 2016).

## Data availability statement

The datasets presented in this study can be found in NCBI SRA repository, accession number PRJNA946556 (https://www.ncbi.nlm.nih.gov/sra/PRJNA946556).

## Author contributions

MY: Data curation, Methodology, Writing – original draft. YaL: Formal analysis, Writing – review & editing. JJ: Software, Writing – review & editing. YoL: Resources, Writing – review & editing. YS: Funding acquisition, Methodology, Resources, Writing – review & editing.
