# Supplementary material for: Gut yeast diversity of Helicoverpa armigera (Lepidoptera: Noctuidae) under different dietary conditions
Source: Front Microbiol. 2024 May 2;15:1287083. doi: 10.3389/fmicb.2024.1287083 (PMC11098133; doi:10.3389/fmicb.2024.1287083)
Supplement: Supplementary file 3 [file Data_Sheet_3.PDF]

Laboratory artificial feeding procedure for cotton bollworms

The fourth-generation eggs were fed with artificial diet to the first instar larvae, and then transferred into three fruits and artificial diet to the third instar larvae. The specific process is as follows.

First, peel the apples and pears, and then cut the apples and pears into small strips that can be put into a small centrifuge tube. The first-instar cotton bollworm was plucked one by one with a brush tip and transferred to a prepared centrifuge tube and a feeding box. A larva and fruit were put into the centrifuge tube and then the mouth of the tube was sealed with cotton to prevent the cotton bollworm larva from climbing out. Place tomatoes and two or three larvae in the bottom of the feeding box after lining paper (Figure 1). Change the fruit once every two days for about five days. Each fruit and artificial diet group was fed with 50 larvae. Each group was repeated three times (30 healthy third instar larvae were selected for each replicate).

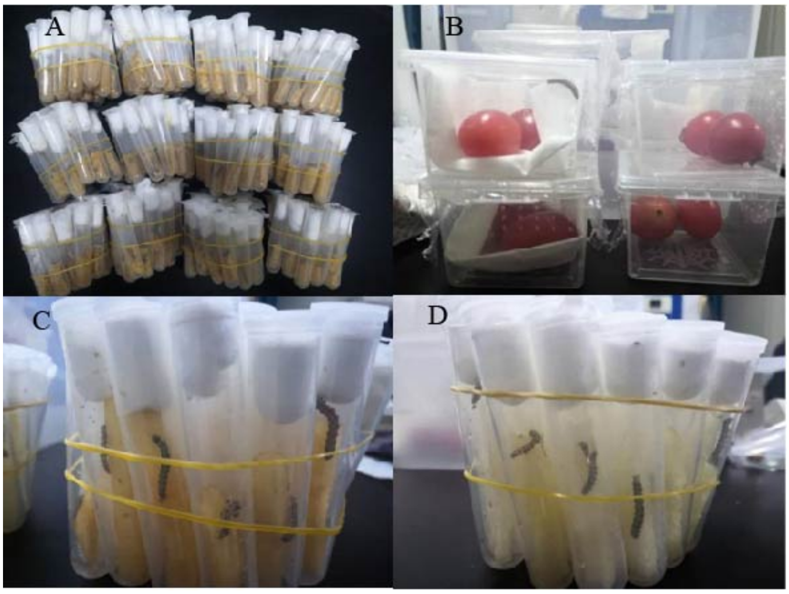

Figure 1. Cotton bollworm in captivity.

Note：A: artificial feed control; B: tomato group; C: apple group; D: pear group.

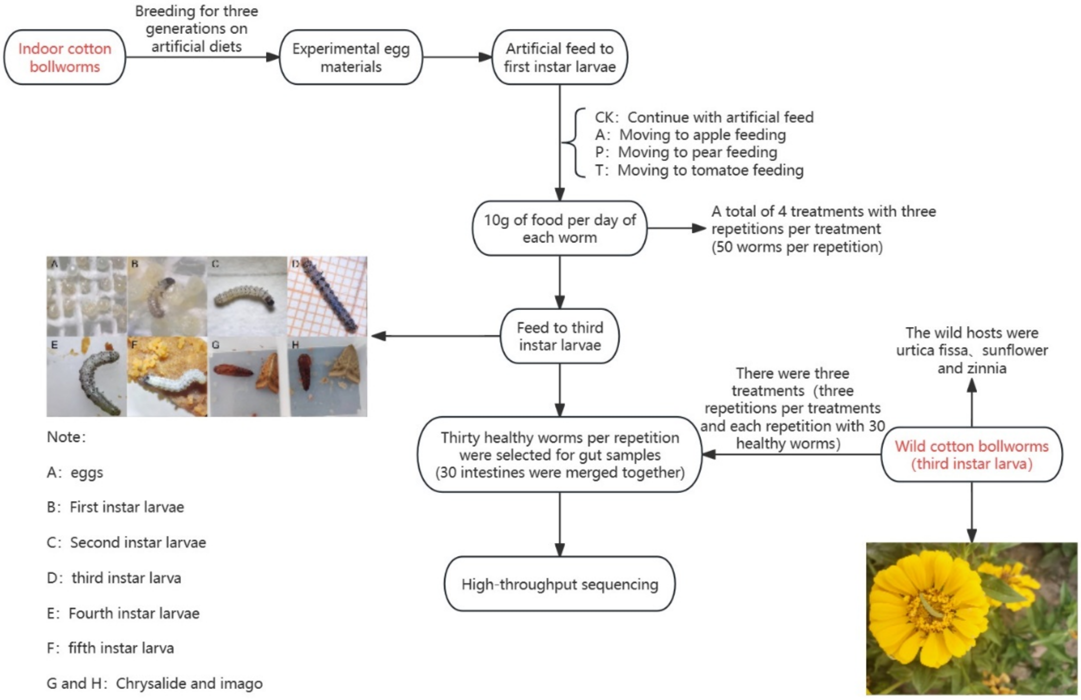

Figure 2. Flowchart of experimental design

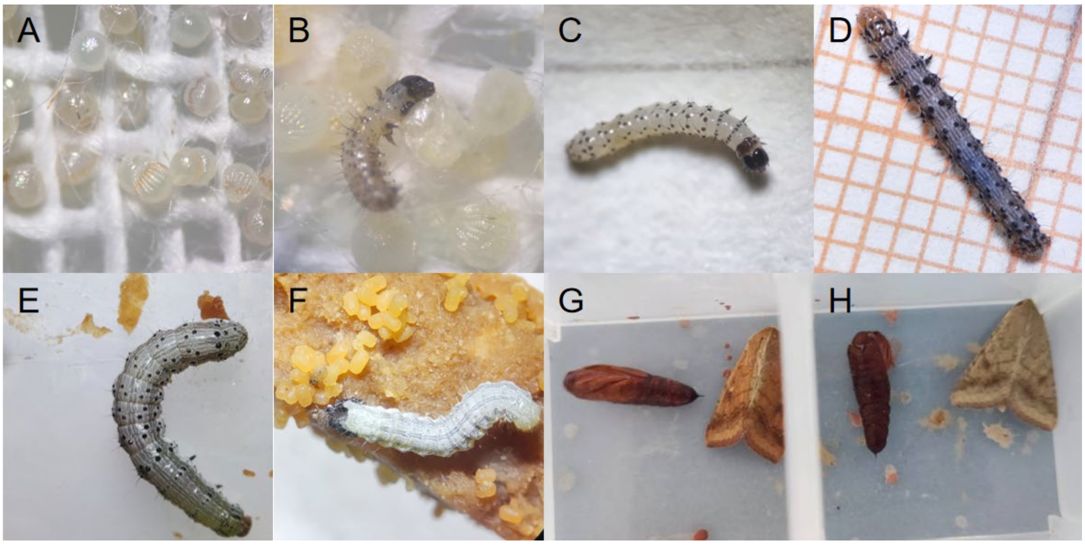

Figure 3. Growth period diagram of cotton bollworm

Note: A: eggs, B: First instar larvae, C: Second instar larvae, D: third instar larva, E: Fourth instar larvae, F: fifth instar larva, G and H: Chrysalide and imago.
